# Basal ganglia hemorrhage in a case report following spinal surgery

**DOI:** 10.1186/s12883-018-1218-x

**Published:** 2018-12-14

**Authors:** Brent Berry, Malik Ghannam, Caitlin Bell, Sami Ghazaleh, Sherief Boss, Christopher Streib, Mustapha Ezzeddine

**Affiliations:** 1 Neurology Department, University of Minnesota, Minneapolis, MN USA; 2 University of Minnesota, Minneapolis, MN USA; 3 Internal Medicine Department, University of Toledo, Toledo, OH USA; 4 Neurosurgery and Radiology, Neurology Department, University of Minnesota, Minneapolis, MN USA

**Keywords:** Spinal surgery, Hemorrhage complications, Supratentorial intraparenchymal hemorrhage, Basal ganglia hemorrhage

## Abstract

**Background:**

Intracranial hemorrhage is a rare but potentially severe complication of spinal surgery. Most reported post-operative ICH cases consist of cerebellar hemorrhage. There are fewer reported cases of supratentorial ICH following spinal surgery.

**Case presentation:**

A 56-year-old woman underwent spinal surgery complicated by bilateral supratentorial intraparenchymal basal ganglia hemorrhage with both intraventricular extension and subarachnoid hemorrhage in both cerebral hemispheres.

**Conclusion:**

The occurrence of neurological deterioration post-operatively following spinal surgery should alert physicians to the possibility of intracranial hemorrhage in order to facilitate rapid and optimal management. To our knowledge, this is the first case reporting basal ganglia hemorrhage following spinal surgery. Moreover, consideration should be given to the possibility of this complication prior to recommendation of elective spinal surgery.

## Background

Intracranial hemorrhage (ICH) is a rare but potentially severe complication of spinal surgery. The first case of post-operative cerebellar hemorrhage was reported by Chadduck in 1981 [1]. Several more authors have since reported cases of cerebellar hemorrhages – and to a lesser extent cerebral hemorrhages – as a delayed complication of spinal surgery [1–5]. Konya et al presented their own case report and conducted a literature review of ten reported cases of remote cerebellar hemorrhage (RCH) [3]. Hempelmann et al. presented three case reports of post-operative cerebellar or cerebral hemorrhage after cerebrospinal fluid (CSF) loss [2]. There was no correlation found between RCH and age, sex, pathology operated, type of intervention performed, or intraoperative body position. All cases had reported significant intraoperative CSF leakage, which is postulated to result in venous infarction due to the downward displacement of the cerebellum and subsequent venous stretching.

For reasons unknown, there have been fewer reported cases of supratentorial intraparenchymal hemorrhages following spinal surgery. Our literature search revealed only two reported cases of supratentorial intraparenchymal hemorrhage. Thomas et al and Morandi et al have each reported a case of cerebral as well as cerebellar hemorrhages after sustaining significant CSF hypotension [4, 5]. Our case is unique in that we present a case of a 56-year-old woman who developed an isolated supratentorial intraparenchymal hemorrhage following spinal surgery. To our knowledge, this is the first case reporting basal ganglia hemorrhage after spinal surgery.

## Case presentation

**Fig. 1:**
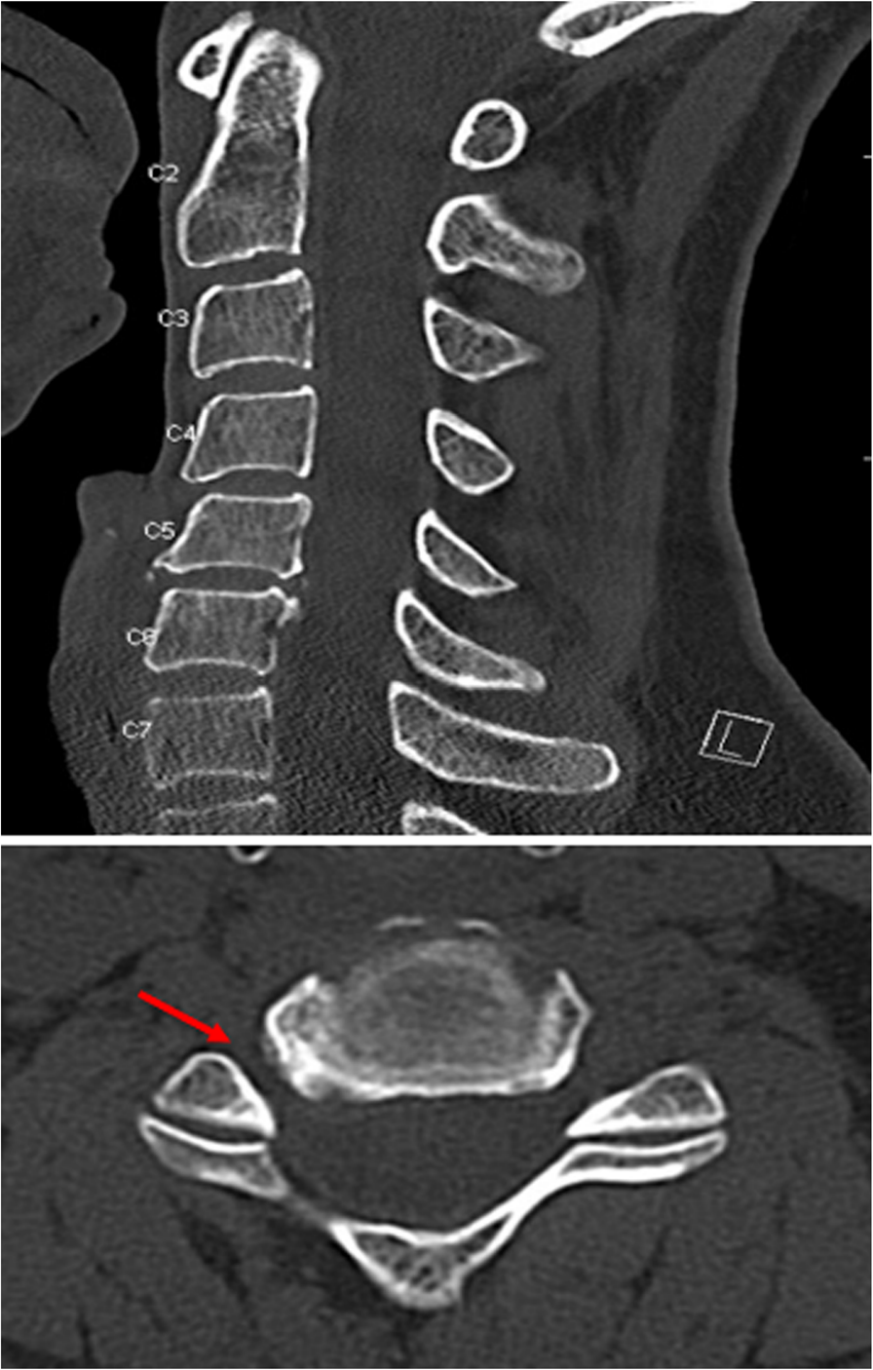
Pre-Operative CT assessment of patient after months-long and progressive C6 radicular symptoms. Moderate cervical spondylosis most pronounced at C4–5 and C5–6. At C5–6, there is considerable right neural foraminal narrowing (red arrow), mild left neural foraminal narrowing and mild spinal canal narrowing

A 56-year-old woman presented with 6 years of radicular symptoms of the right upper extremity. Over the previous year she experienced progressive neck pain with radiation into the right hand in a C6 and C7 distribution. Six years prior she had undergone a posterior cervical laminotomy and foraminotomy without complete resolution of her symptoms. On examination of the right upper extremity, she had normal strength of the deltoid and wrist extensors with reduced strength of the biceps, triceps, and wrist flexors. She also had a positive Spurling’s sign on the right and diminished sensation of the C6 and C7 dermatomes. Her left upper extremity had normal strength and sensation. A cervical computed tomography imaging (CT) showed broad-based bulging of the C5-C6 disc and uncal hypertrophy causing severe right-sided foraminal stenosis [Fig. 1].

**Fig. 2:**
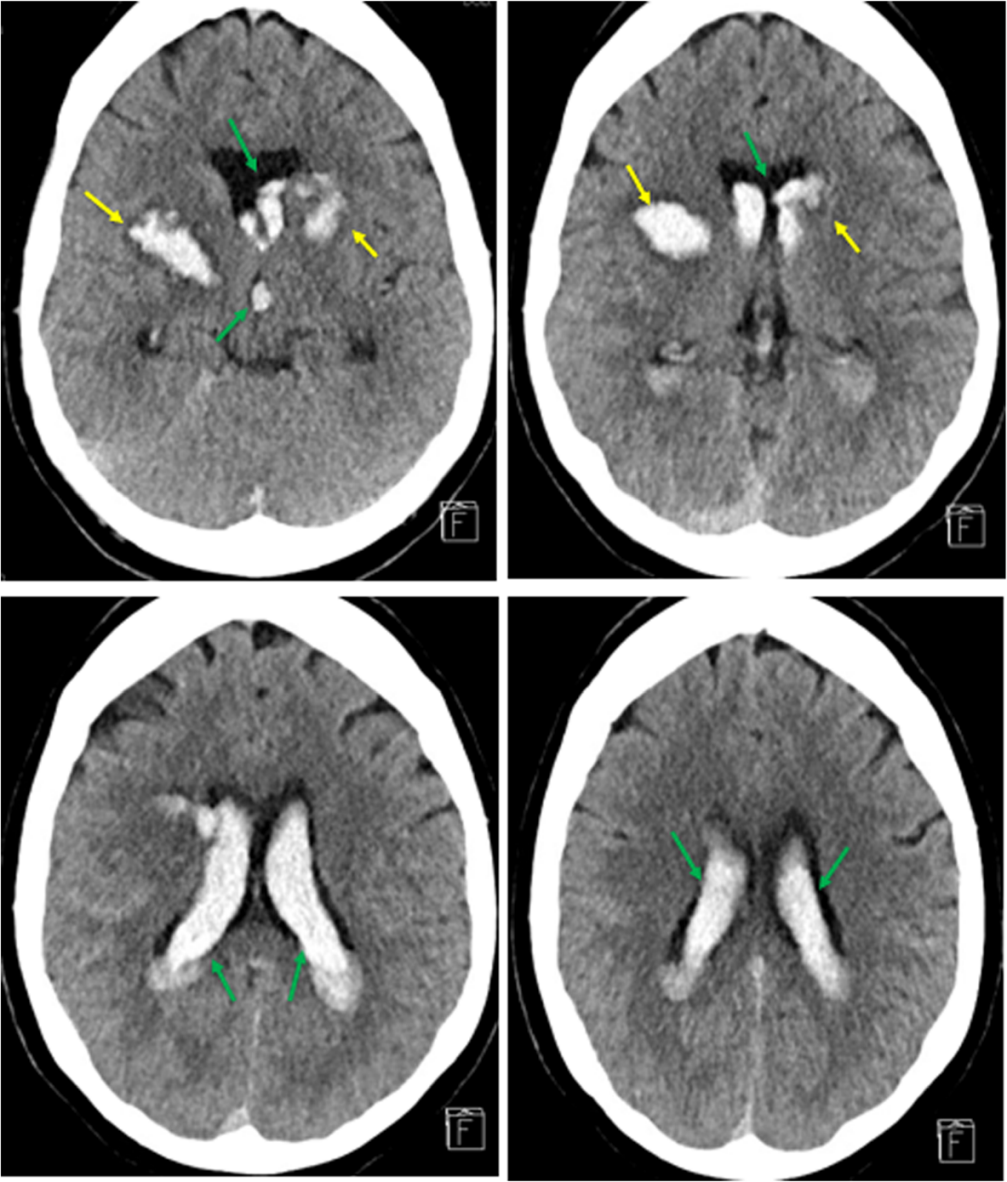
Bilateral Intraventricular Hemorrhage after spinal surgery in the PACU. Large intraventricular hemorrhage within the bilateral lateral ventricles which extends into the third and fourth ventricle and towards the spinal canal (green arrows). Bilateral evolving basal ganglia hemorrhage (yellow arrows). On the right measuring 12 × 23 × 18 mm. On the left measuring 16 × 13 × 13 mm. This is unchanged since 12/6/2017. No ventricular enlargement. Small subarachnoid hemorrhage in the bilateral cerebral hemispheres, primarily along the bilateral parietal convexities. No evidence of midline shift, or abnormal extra-axial fluid collection and maintained gray-white differentiation

Upon consultation with neurosurgery, she elected to pursue the spine surgery. The morning prior to the surgery, her platelet count was 189, INR was 1.05, PTT was 28, bleeding time was within normal limits. A standard Smith-Robertson approach was used to access the anterior spine from the patient’s left side for an anterior cervical discectomy, removal of osteophytes at C5 and C6, prosthetic disc replacement, bilateral foraminotomies at C5 and C6, and an anterior C5 and C6 spinal fusion. Patient was induced with propofol and re-positioned at which point there was a blood-pressure elevation of 155/98 which corrected within five minutes to baseline. Upon awakening in the post-anesthesia care unit, she was found to have altered mental status and was minimally responsive to verbal and sternal stimuli. She could follow some commands with her right arm and leg but was unable to move her left upper or lower extremities. A computed tomography (CT) scan showed bilateral acute basal ganglia hemorrhages with intraventricular extension of the hemorrhage and a small amount of subarachnoid hemorrhage within both cerebral hemispheres [Fig. 2]. It is worth mentioning that the patient had minimal medical problems prior to this surgery and notably was without hypertension, known amyloid angiopathy, or diabetes.

**Fig. 3:**
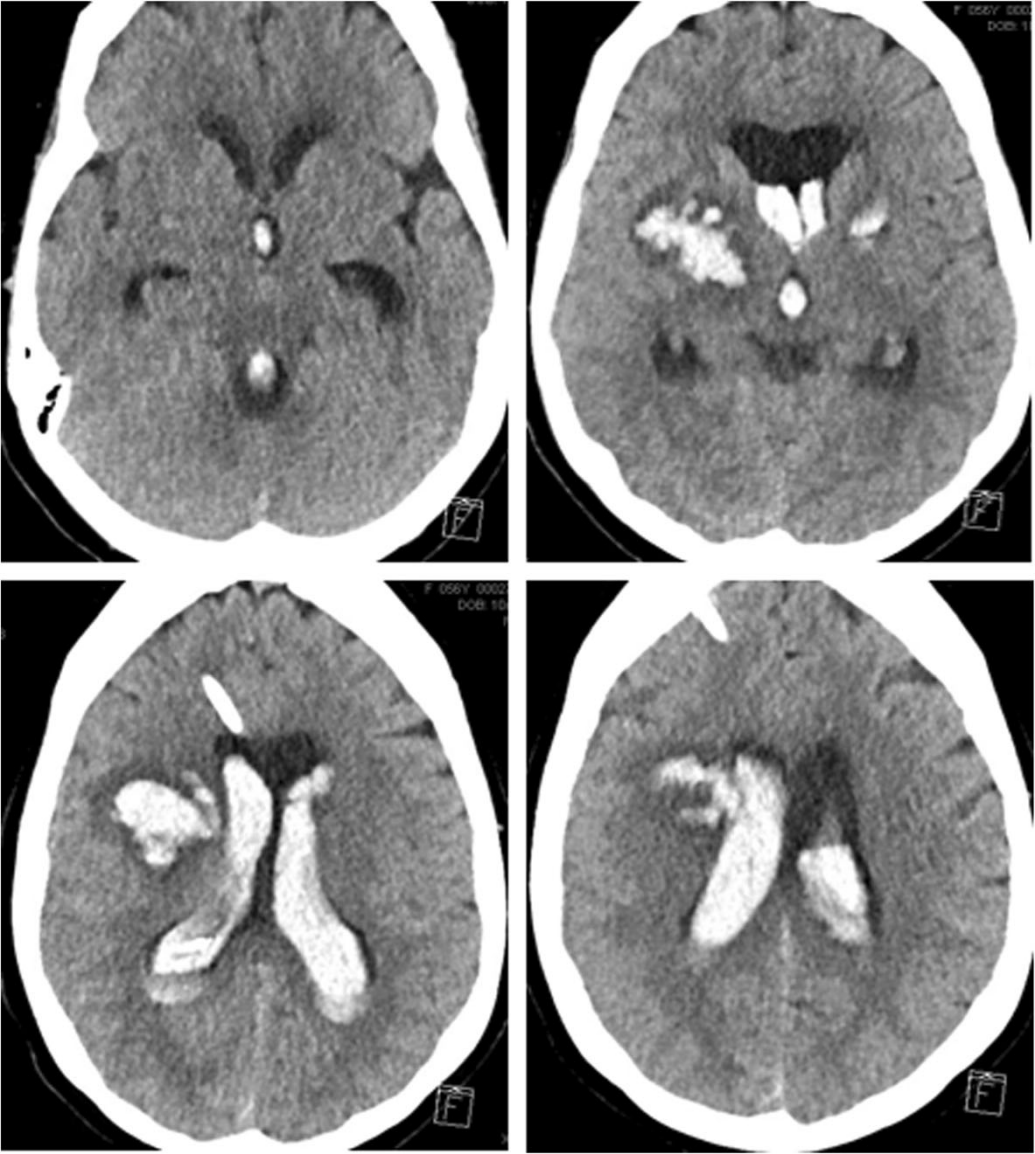
Expanded Bilateral Intraventricular Hemorrhage 24 h post-surgery. Increase in size of 3.1 × 2.4 cm hyperdense right basal ganglia/thalamic intraparenchymal hematoma, previously 2.4 × 1.6 cm. Stable approximately 1.2 × 1.7 cm focus of intracranial hemorrhage in the left basal ganglia. Basal ganglia hemorrhages demonstrate surrounding vasogenic edema and intraventricular extension of hemorrhage. Increased moderate diffuse intraventricular hemorrhage. Stable small bilateral perisylvian region and posterior fossa subarachnoid hemorrhage. No midline shift or herniation

She was immediately transferred to the neurocritical care unit, where she subsequently underwent a diagnostic cervicocerebral angiogram that was normal. Platelet function assays assessed after the surgery were without abnormalities. Twenty-four hours later, the patient acutely declined after an episode of non-bloody emesis and several generalized tonic-clonic seizures. On physical examination, she had anisocoria – left pupil was 5 mm and right pupil was 4 mm – with extensor posturing of the right upper and lower extremities. A computed tomography (CT) brain scan showed an expanding intraparenchymal hematoma of the right basal ganglia [Fig. 3]. She was emergently intubated and underwent emergent placement of an external ventricular drain (EVD). She was extubated one week later. Over the next several weeks she failed numerous EVD clamping trials due to intracranial pressures rising greater than 20 mmHg. She was scheduled for placement of a ventriculoperitoneal shunt; however, this was ultimately aborted after she developed ventriculitis due to colonization with *Propionibacterium acnes*. Throughout this course she gradually developed a left facial droop, mildly dysarthric speech, left visual neglect, and a right gaze preference.

After almost 4 weeks in the neuro-ICU, the patient eventually regained spontaneous movements of her right upper and lower extremities. However, she continued to have extensor posturing in the left upper extremity and triple flexion in the left lower extremity. Sensation was intact throughout all extremities. Serial CT brain scans showed a decrease in the size of the bilateral basal ganglia and intraventricular hemorrhages. She was discharged in this condition to an acute rehabilitation unit 30 days after her elective spinal surgery. During the course of her rehabilitation, her aphasia and right hemiparesis recovered substantially, however, she continued to have profound left hemiparesis three months post-operatively. She requires an AFO and wrist extension brace on her left side and primarily uses a wheel chair.

## Discussion

We describe a case of intracranial hemorrhage involving bilateral basal ganglia (Globus pallidus and putamen) with intraventricular hemorrhage (IVH) and subarachnoid hemorrhage (SAH) following a C5/6 anterior cervical disk replacement. Intracranial hemorrhage after spinal surgery is extremely rare and only a few cases have been described in the literature.

Several risk factors have been associated with spontaneous intracranial hemorrhage including hypertension, amyloid angiopathy, vascular malformations, the use of anticoagulants, tumors, trauma, and infection [6]. The etiology of postoperative intracranial hemorrhage, however, is not well understood. Some have postulated that it might be related to dural tears or drain placement during spinal surgery. This may lead to excessive cerebrospinal fluid (CSF) loss decreasing intracranial CSF pressure. As a result, the bridging veins may stretch and tear causing intracranial bleeding [2, 7]. In our case, it is worth noting that the patient did not have history of hypertension and there were two blood pressure elevations in the pre/perioperative period although the clinical significance of this cannot be fully determined and to some extent is routine course in any major surgery. Prior to the surgery, the patient’s blood pressure was steadily within the ranges 110–126/70–82. Shortly after induction, the patient had an elevation of blood pressure to 155/96 then a return to baseline over the next 10 min with another spike near time of first incision (about five minutes later) to 150/100 with gradual return to baseline range occurring within 10 min. Over the next 24 h blood pressure ranged 104–132 systolic and 68–90 diastolic.

The recognition of CSF leak or dural tear was not noted by surgical members of the team during the patient’s care. However as is noted by Andrews & Koci, 1995; Farag et al., 2005; Friedman et al., 2002; Khatlabari et al., 2012; Miglis & Levine, [10]; and You et al., 2012, CSF leak can be non-obvious at time of surgery and may still be a cause of hemorrhage in such cases [8]. It is possible in some cases for a dural tear not to result in overt rupture of the arachnoid membrane and thus may not necessarily lead to CSF leak. This area is sensitive however particularly when instrumenting and introducing more dead space less liable to tamponading effects of nearby musculature, to induce small dural tears. Even through increasing intra-abdominal pressure which is common during anesthesia, can such a thing occur. So while no CSF leak was noted during surgery, it is possible there was leak.

Cerebellar hemorrhage (CBH) accounts for the vast majority of postoperative intracranial hemorrhage [1, 9, 10]. Other forms of postoperative bleeding include intracerebral hemorrhage (ICH) [2, 4, 11], subdural hemorrhage (SDH) [12, 13], SAH [14], and IVH [8, 12, 15–17]. Depending on the location of the hemorrhage, patients may present with altered level of consciousness, headache, nausea, vomiting, dysarthria, ataxia, and motor or sensory deficits [8].

Intraventricular hemorrhage following spinal surgery is similarly rare with our literature search producing only five reported cases. These five cases share the occurrence of intraoperative dural tear and possible CSF loss. Kim et al. reported a 56-year-old woman who developed bilateral CBH, SAH, and fourth ventricular hemorrhage following a spinal interbody fusion for the treatment of a herniated lumbar disc at L4-L5 and congenital spondylolisthesis [15]. Khalatbari et al. described a 75-year-old man who developed bilateral CBH and fourth ventricular hemorrhage after undergoing lumbar laminectomy for a lumbar stenosis from L1 to L5 [12]. Kaloostian et al. reported a 77-year-old man who was found to have bilateral CBH and fourth and lateral ventricular hemorrhages following a T11–S1 posterior instrumented fusion with an L2 pedicle subtraction osteotomy for kyphoscoliosis and ankylosing spondylitis [8]. Lee et al. described a 76-year-old woman who developed hemorrhage in the cerebral aqueduct and fourth ventricle resulting in obstructive hydrocephalus after she underwent L3-S1 laminectomies and fusion for lumbar spondylosis and stenosis [16]. Guryildirim et al. reported a 76-year-old woman who developed isolated IVH in the third ventricle, cerebral aqueduct and the fourth ventricle following L3-S1 laminectomies and posterolateral instrumented fusion for spinal stenosis [17]. Our report is unique in that we present a case of a 56-year-old woman who developed an isolated supratentorial intraparenchymal hemorrhage following spinal surgery, to our knowledge, this is the first case reporting basal ganglia hemorrhage after spinal surgery.

## Conclusion

Intracranial hemorrhage is a known complication following spinal surgery, especially if the operation is complicated by dural tear and CSF leak. The occurrence of neurological deterioration during the postoperative period should alert physicians to the possibility of intracranial hemorrhage. Emergent neuroimaging should be performed to exclude the diagnosis and facilitate rapid intervention. To our knowledge, this is the first case reporting basal ganglia hemorrhage after spinal surgery. Additionally, consideration should be given to the possibility of this complication prior to recommendation of elective spinal surgery.

## Abbreviations

AFO: Ankle Foot Orthosis
CBH: Cerebellar Hemorrhage
CSF: Cerebrospinal Fluid
CT: Computed Tomography
EVD: External Ventricular Drain
ICH: Intracerebral Hemorrhage
ICU: Intensive Care Unit
IVH: Intraventricular Hemorrhage
RCH: Remote Cerebellar Hemorrhage
SAH: Subarachnoid Hemorrhage
SDH: Subdural Hemorrhage
